# Exhaled air profile in the early diagnosis of ventilator-associated pneumonia

**DOI:** 10.62675/2965-2774.20240194-en

**Published:** 2024-10-07

**Authors:** Rodrigo Cruvinel Figueiredo, Jackelyne Lopes Silva, Igor Bianchini, Luana Bezerra Gonçalves Rocha, Renata Casagrande Goncalves, Cristiane Ritter, Felipe Dal-Pizzol

**Affiliations:** 1 Centro Universitário do Espírito Santo Colatina ES Brazil Centro Universitário do Espírito Santo - Colatina (ES), Brazil.; 2 Universidade do Extremo Sul Catarinense Criciúma SC Brazil Universidade do Extremo Sul Catarinense - Criciúma (SC), Brazil.

**Keywords:** Pneumonia, ventilator-associated, Respiration, artificial, Inpatients, Electronic nose, Gases, Volatile organic compounds, Reference standards, Predictive value of tests, Mechanics, Intensive care units

## Abstract

**Objective:**

To predict exhaled air in patients undergoing mechanical ventilation during bedside diagnosis of ventilator-associated pneumonia.

**Methods:**

Air samples were collected through the expiratory branch of the mechanical ventilation circuit during the hospitalization of patients at the intensive care unit of Hospital São José in Criciúma (SC), Brazil. In this study, 83 participants were divided into two groups, namely, the group with and the group without ventilator-associated pneumonia.

**Results:**

The analysis of three air patterns revealed a predictive value for the diagnosis of ventilator-associated pneumonia. The analyses of samples from the first 12 hours of invasive mechanical ventilation were able to predict ventilator-associated pneumonia (p = 0.018). However, none of the other air samples collected during hospitalization were useful in identifying the severity or predicting early or late ventilator-associated pneumonia.

**Conclusion:**

The use of a gas analyzer may be helpful for the early identification of patients admitted to intensive care who will develop ventilator-associated pneumonia.

## INTRODUCTION

The incidence of ventilator-associated pneumonia (VAP) has decreased in recent years. According to the Centers for Disease Control and Prevention (CDC), the incidence of VAP in intensive care units is approximately 0.9 per 1,000 days of mechanical ventilation. The current definition of VAP includes subjective qualitative criteria, such as worsening of the appearance and amount of tracheal secretion, as well as criteria for worsening oxygenation. It is not yet known whether the decrease in registered VAP cases reflects the application of these subjective diagnostic criteria.^(1)^

Recent studies have shown that the accuracy of the clinical diagnosis of VAP is low, and this factor contributes to the indiscriminate use of antibiotics in the intensive care environment. Symptoms such as fever and purulent secretion had a sensitivity and specificity of 66.4% and 53.9%, respectively. Radiological images and culture samples of tracheal secretions have a sensitivity of approximately 74% and specificity of 56%.^(2)^

To make a definitive and earlier diagnosis, a new methodology is still needed. For some time, the analysis of volatile organic compounds (VOCs) present in the exhaled air of the lungs has gained importance in the determination of biomarkers for screening, diagnosing and monitoring the treatment of oncological, inflammatory, infectious and even psychiatric diseases.^(3,4)^ Exhaled VOCs are typically analyzed by state-of-the-art laboratory tools, such as gas chromatography‒mass spectrometry (GC-MS), field asymmetric ion mobility spectrometry (FAIMS), or electronic nose-type recognition sensors.^(5,6)^ The latter method involves the interaction of chemical sensors and the volatile organic components released during expiration. The generation of different signature groups after pattern recognition algorithms and statistical models leads to the classification of odors as “compatible” or “noncompatible”.^(7-9)^

Considering the lack of a single and efficient method, the purpose of this study was to evaluate the use of the exhaled air profile for the early detection and prediction of VAP in patients admitted to the intensive care unit (ICU).

## METHODS

### Patients

A prospective cohort study was conducted at the Intensive Medicine Service of *Hospital São José* in Criciúma (SC), Brazil, including patients aged ≥ 18 years and who underwent invasive mechanical ventilation for 48 hours or more. Patients with diseases known to affect the quality of exhaled air (other types of parenchymal lung diseases, such as cystic fibrosis, idiopathic pulmonary fibrosis and bronchiectasis) or other clinical conditions still unknown to affect air quality when exhaled but could interfere with the immune response or lead to the accumulation of metabolites (immunodeficiencies, severe hepatic or renal failure requiring replacement dialysis therapy).

The study was conducted in accordance with the national and international resolutions described in Resolution 466 of October 12, 2012, and with the complementary resolutions of the National Council of ^Health^/Ministry of Health, in addition to the Declaration of Helsinki and all its revisions and amendments. The study was approved by the Research Ethics Committee of the *Universidade do Extremo Sul Catarinense*. with the CAAE protocol: 07789019.0.0000.5364. The data obtained from the medical records were kept confidential by the investigators, with the guarantee of anonymity for all the data collected. The informed consent form of the participants was waived by the local Research Ethics Committee because the study was purely observational.

### Procedures

The first measurement of the exhaled air profile was performed during the first 12 hours of mechanical ventilation installation. The remaining measurements were performed daily, once a day, for up to 3 days after the diagnosis of VAP or complete weaning from mechanical ventilation, whichever occurred first.

The collection of exhaled air was performed aseptically by coupling the needle of the gas analyzer device to the expiratory route of the mechanical ventilator. Depending on the type of mechanical ventilation device used, this collection was performed immediately after the bacteria passed through the bacterial filter located in the “Y” of the circuit or directly after the filter located next to the exhalation valve of the device. The time required for analysis and air capture was automatically signaled by the display of the electronic nose device via audible and visual signals.

All the results of the collected samples were stored throughout the study collection period in an appropriate spreadsheet in the software provided by the device manufacturer for further data analysis. The collected samples were acquired with the device calibrated with room air. This allowed the comparison of the different patterns of VOC classes found in relation to a “pure” air sample.

Data during the entire data collection period, demographic data, data on the presence of preexisting chronic diseases, and data on the prognostic scores (Simplified Acute Physiology Score 3 [SAPS 3] and Sequential Organ Failure Assessment [SOFA]) were recorded and other relevant clinical information of the patients was included in the study.

The diagnosis of VAP was determined according to the criteria used in the intensive care unit of the Hospital São José. This criterion followed the VAP diagnosis flowchart established by the National Health Surveillance Agency (ANVISA - *Agência Nacional de Vigilância Sanitária*), and all diagnoses of the patients included in the study were endorsed by the Hospital Infection Control Committee of the hospital itself.^(10)^

### Gas analyzer

The technology applied to capture and identify the VOCs in the study was Cyranose^®^ 320. It is a small, commercially available, portable electronic nose with 32 channels that can be used at the bedside in a noninvasive manner. The device allows for rapid analysis of exhaled gases, as it has odor learning technology and may be useful in identifying specific patterns related to VAP.^(11-14)^ The gas analyzer does not determine exactly which VOC is present in each of the samples; it has 32 electrochemical sensors that detect exhaled air patterns (each sensor reacts with the VOCs present in the air, generating an electrical signal). The data from these 32 sensors are then reduced via principal component analysis (PCA) to the main components of each collection by means of the software of the device. These PCA components were used to determine exhaled air patterns. For this study, the exhaled air profiles were called patterns 1, 2 and 3.

### Statistical considerations

The statistical considerations were based on studies using Cyranose^®^ 320 in other lung diseases, which estimated an incidence of VAP of 35% in air pattern 1 and 15% in air pattern 2, with a power of 80% and a significance of 0.05, resulting in a sample size of 70 patients.

Primary analysis was performed to compare the exhaled air profile between patients with and without VAP. For the primary analysis, the first air collected after inclusion in the study, the air collected 24 hours before the clinical diagnosis of VAP and the change in the air profile between these two times were considered. This period was selected considering the hypothesis that early changes in the exhaled air profile may predict the subsequent development of VAP. For the Non-VAP Group, the air collected 96 hours after the start of mechanical ventilation was considered for comparison purposes with the air collected 24 hours before the diagnosis of VAP. This period was selected to compare the profiles with similar durations of mechanical ventilation in the Control Group (No VAP).

A secondary analysis was performed only in patients with VAP to determine the ability of the exhaled air profile to predict clinically relevant outcomes (time on mechanical ventilation and length of stay in the ICU). Therefore, in addition to the collections mentioned above, data from the air collected on the day after this diagnosis were used. Thus, it was possible to determine not only the predictive value of cross-sectional exhaled air collection but also the impact of the temporal change in the exhaled air profile and its relationship with the outcome.

Continuous variables are expressed as the means and standard deviations (SDs). Categorical data are expressed as frequencies and percentages. The groups were compared via the Kruskal‒Wallis test and the Wilcoxon‒Mann‒Whitney test for continuous outcomes, and the chi‒square test was used for categorical outcomes. Multivariate binary logistic regression was used to analyze the adjusted differences between the groups. All variables with p values < 0.25 in the univariate analysis, in addition to age, sex, SAPS 3 score and SOFA score in the first 24 hours, were incorporated into the model. The regression results are expressed as the relative risk (RR) and interquartile range (IQR). In all analyses, a p value < 0.05 was adopted as the level for statistical significance.

## RESULTS

**Figure 1:**
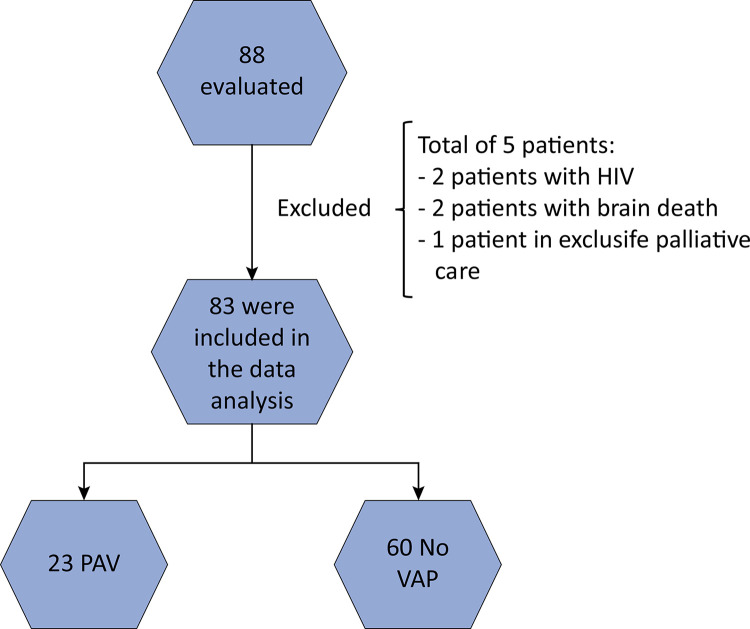
Study participants.

During the study period, 88 patients were evaluated, and with respect to the predefined exclusion criteria, 83 patients were included in the final analysis (Figure 1); among them, 38 (46%) had microbiological confirmation. The median duration of VAP development was 4 days, with a 25 - 75 interval between Days 4 and 6.

VAP - ventilator-associated pneumonia.

In the comparison between the VAP Group and the Non-VAP Group, there was no significant difference between the main characteristics of the patients at the time of inclusion: mean age of 60 years (p = 0.29), male predominance in both groups (p = 0.66), patients hospitalized mainly for medical reasons (p = 0.21), SAPS 3 severity score with a mean score of 52.5 (p = 0.38) and SOFA organ dysfunction score with a mean score of 8, 4 (p = 16) (Table 1). In the binary regression, none of these variables were independently associated with the occurrence of VAP. Among the sociodemographic and clinical characteristics of the patients who presented patterns 1, 2 or 3 of exhaled air, there were also no differences in the variables analyzed (Table 2).

**Table 1:** Characteristics of the patients at the time of inclusion in the study

Variables	PAV Group (n = 23)	Non-VAP Group (n = 60)	p value	RR (IQR)
Age (years)	58 ± 16	62 ± 15	0.29	
Male sex	15 (65.2)	36 (60)	0.66	
Reason for hospitalization				
Clinical	14 (60)	30 (50)		REF
Urgent surgery	8 (34.7)	18 (30)	0.21	0.52 (0.15 - 1.7)
Elective surgery	1 (4.3)	12 (20)		1.43 (0.15 - 1.3)
Comorbidities				
None	8 (34.7)	19 (31.6)		
Pulmonary	2 (8.6)	6 (10)	0.95	
Others*	13 (56.5)	35 (58.3)		
SAPS	50 ± 18	55 ± 20	0.38	
SOFA D1	7.9 ± 2.6	9 ± 3.5	0.16	0.85 (0.71 - 1,01)

VAP - ventilator-associated pneumonia; RR - relative risk; IQR - interquartile range; SAPS - Simplified Acute Physiology Score; SOFA - Sequential Organ Failure Assessment score; D1 = Day 1. * Cardiovascular diseases and diabetes. The results are expressed as the means *±* standard deviations or n (%).

**Table 2:** Characteristics of the patients in relation to each air pattern found in the first 12 hours after admission in the VAP and Non-VAP Groups

Variables	Pattern 1	Pattern 2	Pattern 3	p value
Age (years)	65 ± 15	57 ± 17	61 ± 12	0.89
Male sex	18 (58)	21 (63)	12 (63)	0.88
Clinical reason for hospitalization	19 (61)	15 (45)	10 (52)	0.30
Pulmonary comorbidities	3 (9.6)	3 (9)	2 (10)	0.84
SAPS	53 ± 22	57 ± 18	48 ± 19	0.29
SOFA D1	8.5 ± 3.4	8.8 ± 3.1	8.8 ± 3.6	0.19

SAPS - Simplified Acute Physiology Score; SOFA - Sequential Organ Failure Assessment Score; D1 - Day 1. The results are expressed as the means *±* standard deviations or n (%).

The air patterns analyzed in the first 12 hours after mechanical ventilation (admission air) were predictors of the development of VAP during hospitalization (p = 0.018), even when they were subjected to binary regression (Table 3). However, the analysis of exhaled gas patterns 24 hours before the clinical diagnosis of VAP (pre-VAP air) was not a predictor of the development of VAP (p = 0.35). There was also no significant change (p = 0.3) (Table 3) in the air patterns analyzed during the participants’ hospitalization. At each of the two times studied (admission air and pre-VAP air), as well as the change in the air pattern reported in some patients, the sensitivity, specificity and positive predictive value for the development of VAP showed few results (sensitivity and specificity, 30% and 60%, respectively, at air admission for patterns 2 and 3; sensitivity and specificity, both 60%, in the air pre-VAP; and sensitivity, 30%; specificity, 75%; and positive predictive value, 39%, for the change in the air standard).

**Table 3:** Relationships between the identified air patterns and the development of ventilator-associated pneumonia

	PAV Group (n = 23)	Non-VAP Group (n = 60)	p value	RR (IQR)	RR (IQR)*
Air in admission†					
Pattern 1	3 (13)	28 (46)		REF	REF
Pattern 2	13 (56)	20 (33)	0.018	8.05 (1.8 - 35)	6.8 (1.6 - 29)
Pattern 3	7 (30)	12 (20)		5.9 (1.2 - 29)	5.8 (1.2 - 27)
Pre-VAP air‡					
Pattern 1	6 (26)	15 (25)			
Pattern 2	11 (47)	11 (18)	0.35		
Pattern 3	6 (26)	8 (13)			
Change§, yes	9 (39)	18 (53)	0.3		

VAP - ventilator-associated pneumonia; RR - relative risk; IQR - interquartile range. * Adjusted for binary regression; † air sample analyzed up to 12 hours after inclusion; ‡ air sample analyzed 24 hours before the clinical diagnosis of VAP; § change in the air pattern during hospitalization of the participants. The results are expressed as n (%).

The mean time of mechanical ventilation and the number of days of ICU stay were higher in the VAP Group than in the control group, as expected. The VAP Group had, on average, 13 days of mechanical ventilation compared to 5 days in the Non-VAP Group (p = 0.0001); the same occurred with the days of ICU stay, with an average of 17 days in the VAP Group and 9 days in the Non-VAP Group (p = 0.0001). Nevertheless, the pattern of exhaled air collected at admission was not related to the duration of MV (p = 0.28) or to the duration of ICU stay (p = 0.40). This was also repeated for air pre-VAP (p = 0.40, p = 0.41) and air after the diagnosis of VAP (p = 0.58, p = 0.23). There was also no significant correlation between a particular pathogen and a specific pattern of exhaled air.

## DISCUSSION

In the present study, we determined that the exhaled air pattern, when collected in the first hours after mechanical ventilation, is able to predict the occurrence of VAP.

Pre-VAP air (air analyzed during the 24 hours before the diagnosis of VAP) was not able to predict VAP as expected by the study authors. However, the analysis of air samples from 12 hours after admission (admission air) was able to predict the diagnosis of VAP. This can be explained by the fact that the development of VAP is related to factors intrinsic to the individual, that is, some characteristics of the study population, not identified in this study, are capable, by themselves, of predicting VAP. Among these characteristics, the degree of inflammation and the quality and intensity of the oxidative stress present in the respiratory mucosa at the time of inclusion of these participants should be highlighted. This early change in air availability may also be explained by the conditions or reasons for which the patient was subjected to intubation. Intubation performed improperly, late or after bronchoaspiration, for example, in theory, may change the mucosa of the airways early. In future studies, this correlation between the immediate reason for orotracheal intubation, whether elective or urgent, and the temporal analysis of exhaled air would be important. This result was also not influenced by the characteristics of the individuals (age, sex, comorbidities, reason for hospitalization, SOFA score, SAPS 3 score and C-reactive protein [CRP] value) when they were distributed in relation to standards 1, 2 or 3 of the studied groups, as there was no difference between these variables among the three groups. VOCs are the result of oxidative stress present in the epithelium of the respiratory tree, and this inflammation may facilitate the development of VAP in this group of patients.^(15)^

Another result corroborated by this discussion was that the air pattern did not change during the follow-up of the participants. In patients who developed VAP, the air pattern remained similar from the beginning of inclusion. Something acquired during hospitalization (e.g., colonization of certain microorganisms already present in the respiratory system, episodes of microaspiration and other risk factors already known in the literature), which may even be related to the development of VAP, does not seem to influence, at least in our patients, the air quality analyzed. The inflammatory characteristics of the respiratory epithelium of the participants at the time of inclusion may have been responsible for the prediction of air admission in the diagnosis of VAP.^(16)^

During all the air analyses performed during the study, the device was calibrated and trained using room air; this method of evaluation may have influenced the results. Cyranose^®^ 320 is a tool that has the ability to learn to detect odors from memory stored in its *software*. This functionality allows the use of the device in the detection of specific or similar odors in various health areas, such as in the early detection of colorectal cancer and in the diagnosis of some pathologies of the respiratory tract, such as asthma and chronic obstructive pulmonary disease.^(4,17)^ If the device is trained with air samples from individuals with a confirmed diagnosis of VAP and air samples from the most common strains of microorganisms in intensive care units, the results may be more predictive and identify in a more refined way a change in the air pattern to characterize an early diagnosis of VAP. This hypothesis may explain why there was no significant change in the air pattern during the evolution of the patients who developed VAP because the device was not trained (calibrated) for the specific detection of similar odors in patients diagnosed with VAP.

Air on admission was able to predict VAP; however, it was not effective in predicting severity in the VAP Group. Although mortality was also expected to be higher in the VAP Group than in the Control Group, it was similar between the groups.^(18,19)^ Consequently, in the population studied, there was no direct relationship between VAP and severity. This was probably due to the small sample size in the VAP Group. There may be a direct relationship between the air analyzed as a predictor of VAP and the same air-predicting severity in a larger population sample. This same justification, the small sample size used in the present study, can be used to explain the low sensitivity and specificity and the positive predictive value.

Similarly, neither admission air nor pre-VAP air was able to distinguish early VAP from late VAP. This may be due to the small sample size in the VAP Group and because the device was calibrated relative to ambient air. It is possible that, in a device trained and calibrated on air samples from individuals with VAP, there is some result in prediction regarding the temporal classification of VAP (early or late).

The results of this study encourage the production of other scientific studies using the electronic nose for the diagnosis of VAP and its microbiological determinants. There are already studies demonstrating the importance of the analysis of exhaled air in some lung infections, namely, in the identification of the microorganism responsible for the infection.^(20)^ The suspicion of the germ that causes VAP at the bedside allows for early antimicrobial treatment and minimizes the indiscriminate use of antibiotics while waiting for the results of respiratory secretions cultures.

Nevertheless, some limitations of the study should be taken into account when the results are analyzed. First, a series of patients with common pathologies in intensive care were excluded, and this reduces the external validity of the study. This limitation results in a more homogeneous patient sample, which may better reflect the influence of mechanical ventilation alone on the development of VAP and minimize the effects of other diseases on the exhaled air profile. Second, owing to limitations of the sample size and the characteristic of the predictor variable (categorical), the construction of a Receiver Operating Characteristic (ROC) curve and its interpretation are limited, hindering a more robust interpretation of the real predictive value of the analysis of exhaled air in the diagnosis of VAP. Third, there is an important difference in the epidemiological definitions used here for the diagnosis of VAP and the clinical criteria for antibiotic initiation. Thus, some patients who, in practice, would be included as having VAP were not included in the present study. The use of the epidemiological definition, on the other hand, makes the VAP Group more homogeneous, which may be important for the primary objective of the study and allows for comparisons and analyses of future results with other studies that used the same defining criterion.

## CONCLUSION

The use of the Cyranose^®^ 320 electronic nose device, through the analysis of exhaled air during the first 12 hours of invasive mechanical ventilation, was able to identify a relationship with the evolutionary development of ventilator-associated pneumonia in the study population. However, the various air samples collected via the electronic nose were not able to predict the severity or identify the development of early or late mechanical ventilation-associated pneumonia among the participants.
