# Supplementary material for: Exhaled air profile in the early diagnosis of ventilator-associated pneumonia
Source: Crit Care Sci. 2024 Oct 7;36:e20240194en. doi: 10.62675/2965-2774.20240194-en (PMC11463982; doi:10.62675/2965-2774.20240194-en)
Supplement: Translation

# Translation: Perfil do ar exalado no diagnóstico precoce de pneumonia associada à ventilação mecânica

**DOI:** 10.62675/2965-2774.20240194-pt

**Authors:** Rodrigo Cruvinel Figueiredo, Jackelyne Lopes Silva, Igor Bianchini, Luana Bezerra Gonçalves Rocha, Renata Casagrande Goncalves, Cristiane Ritter, Felipe Dal-Pizzol

**Affiliations:** 1 Brasil Centro Universitário do Espírito Santo - Colatina (ES), Brasil.; 2 Brasil Universidade do Extremo Sul Catarinense - Criciúma (SC), Brasil.

**Keywords:** Pneumonia associada a ventilação, Respiração artificial, Pacientes internados, Nariz eletrônico, Gases, Compostos orgânicos voláteis, Padrões de referência, Valor preditivo dos testes, Mecânica, Unidades de terapia intensiva

## Abstract

**Objetivo:**

Demonstrar a predição do ar exalado dos pacientes submetidos à ventilação mecânica no diagnóstico à beira leito da pneumonia associada à ventilação mecânica.

**Métodos:**

Foram coletadas amostras de ar através do ramo expiratório do circuito de ventilação mecânica ao longo da internação de pacientes na unidade de terapia intensiva do Hospital São José, em Criciúma (SC). O trabalho analisou 83 participantes subdivididos em dois grupos, com e sem pneumonia associada à ventilação mecânica.

**Resultados:**

Por meio da análise de três padrões de ar identificados, conseguiu-se um valor preditivo para diagnóstico de pneumonia associada à ventilação mecânica. As análises das amostras das primeiras 12 horas de ventilação mecânica invasiva foram capazes de predizer a pneumonia associada à ventilação mecânica (p = 0,018). Entretanto, nenhuma das outras amostras de ar coletadas ao longo da internação dos pacientes foi capaz de caracterizar gravidade ou predizer, em precoce ou tardia, a pneumonia associada à ventilação mecânica.

**Conclusão:**

A utilização de um aparelho analisador de gases pode ser útil na identificação precoce dos pacientes que desenvolverão pneumonia associada à ventilação mecânica internados em terapia intensiva.

## INTRODUÇÃO

A pneumonia associada à ventilação mecânica (PAV) vem apresentando declínio em sua incidência nos últimos anos. De acordo com o *Centers for Disease Control and Prevention* (CDC), dos Estados Unidos, a incidência de PAV nas unidades de terapia intensiva encontra-se em torno de 0,9 por 1.000 dias de ventilação mecânica. A definição atual de PAV inclui critérios qualitativos subjetivos, como piora do aspecto e da quantidade da secreção traqueal, assim como critérios de piora de oxigenação. Ainda não se sabe se a diminuição dos casos de PAV registrada reflete a aplicação desses critérios diagnósticos subjetivos.^(1)^

Estudos recentes mostram que a acurácia no diagnóstico clínico de PAV é baixa, e esse fator contribui para o uso indiscriminado de antibióticos no ambiente de terapia intensiva. Sintomas como febre e secreção purulenta apresentam sensibilidade e especificidade de 66,4 e 53,9% respectivamente. Imagens radiológicas e amostras de cultura de secreção traqueal apresentam sensibilidade em torno de 74% e especificidade de 56%.^(2)^

Com o propósito de realizar um diagnóstico definitivo e mais precoce, uma nova metodologia ainda é necessária. Há algum tempo, a análise de compostos orgânicos voláteis (COVs) presentes no ar exalado dos pulmões vem ganhando importância na determinação de biomarcadores para rastreio, diagnóstico e monitoração do tratamento de doenças oncológicas, inflamatórias, infecciosas e até psiquiátricas.^(3,4)^Os COVs exalados são tipicamente analisados por ferramentas laboratoriais de ponta, como na espectrometria de massa acoplada à cromatografia gasosa (GC-MS - *gas chromatography mass spectrometry*), na espectrometria de mobilidade (FAIMS - *field asymmetric ion mobility spectrometry*), ou por sensores de reconhecimento do tipo nariz eletrônico.^(5,6)^O funcionamento desse último método consiste na interação dos sensores químicos e dos componentes orgânicos voláteis liberados durante a expiração. A geração de diferentes grupos de assinatura após o reconhecimento de padrões de algoritmos e modelos estatísticos leva a uma classificação dos odores como “compatíveis” ou “não compatíveis”.^(7-9)^

Considerando a não existência de um método único e eficiente, o propósito deste estudo foi avaliar o perfil do ar exalado na detecção precoce e na predição de PAV em pacientes internados em unidade de terapia intensiva (UTI).

## MÉTODOS

### Pacientes

Foi realizada uma coorte prospectiva, no Serviço de Medicina Intensiva do Hospital São José, em Criciúma (SC), que incluiu pacientes com idade ≥ 18 anos e que foram submetidos à ventilação mecânica invasiva por 48 horas ou mais. Foram excluídos do estudo os pacientes com doenças que sabidamente alteram a qualidade do ar exalado (outros tipos de doenças pulmonares parenquimatosas, como, por exemplo, fibrose cística, fibrose pulmonar idiopática e bronquiectasias) ou outras situações clínicas ainda desconhecidas em alterar a qualidade do ar exalado, mas que poderiam interferir na resposta imune ou levar ao acúmulo de metabólitos (imunodeficiências, insuficiência hepática grave ou renal com demanda de terapia dialítica substitutiva).

O estudo foi conduzido em conformidade com as resoluções nacionais e internacionais como descritas na resolução 466, de 12 de outubro de 2012 e complementares do Conselho Nacional de Saúde/Ministério da Saúde, além da Declaração de Helsinque e de todas suas revisões e alterações. Foi aprovado pelo Comitê de Ética e Pesquisa da Universidade do Extremo Sul Catarinense com protocolo CAAE: 07789019.0.0000.5364. Os dados obtidos do prontuário médico foram mantidos em caráter confidencial pelos investigadores, com a garantia de anonimato de todos os dados coletados. Foi dispensado, pelo Comite de Ética e Pesquisa local, a coleta do Termo de Consentimento Livre e Esclarecido dos participantes pelo entendimento de o estudo ser meramente observacional.

### Procedimentos

A primeira medida do perfil do ar exalado foi feita durante as primeiras 12 horas de instalação da ventilação mecânica. As demais medidas foram realizadas diariamente, uma vez ao dia, até 3 dias após o diagnóstico de PAV ou o completo desmame da ventilação mecânica - o que ocorresse primeiro.

A coleta do ar exalado foi realizada de forma asséptica, pelo acoplamento da agulha do aparelho analisador de gases na via expiratória do ventilador mecânico. Essa coleta, a depender do tipo de aparelho de ventilação mecânica utilizado, foi feita logo após a passagem do ar pelo filtro bacteriológico presente no “Y” do circuito ou diretamente após o filtro presente próximo à válvula exalatória do aparelho. O tempo necessário de análise e captação do ar era sinalizado de forma automática pelo próprio *display* do aparelho do nariz eletrônico, por sinal sonoro e visual.

Todos os resultados das amostras coletadas foram armazenados, durante todo o período de coleta do estudo, em planilha apropriada no próprio *software* fornecido pelo fabricante do aparelho, para posterior análise dos dados. As amostras coletadas foram adquiridas com o aparelho calibrado com ar ambiente. Isso permitiu a comparação dos diferentes padrões de classes de COVs encontrados em relação a uma amostra “pura” de ar.

Durante todo o período de coleta dos dados, foram registrados os dados demográficos, a presença de patologias crônicas preexistentes, o escore prognóstico (*Simplified Acute Physiology Score 3* [SAPS 3] e *Sequencial Organ Failure Assessment* [SOFA]) e outras informações clínicas relevantes dos pacientes incluídos no estudo.

O diagnóstico de PAV foi determinado pelo critério utilizado no Serviço de Medicina Intensiva do Hospital São José. Esse critério obedecia ao fluxograma de diagnóstico de PAV estabelecidos pela Agência Nacional de Vigilância Sanitária (ANVISA), e todos os diagnósticos dos casos incluídos no estudo foram endossados pela Comissão de Controle de Infecção Hospitalar do próprio hospital.^(10)^

### Analisador de gases

A tecnologia aplicada para captação e identificação dos COVs no estudo foi o Cyranose^®^ *320.* É um nariz eletrônico portátil, de pequeno porte, disponível para comercialização, dotado de 32 canais, que pode ser utilizado à beira do leito de maneira não invasiva. O aparelho permite a análise rápida dos gases exalados, já que possui uma tecnologia de aprendizado dos odores e pode ser útil na identificação de padrões específicos relacionados à PAV.^(11-14)^ O analisador de gases não determina exatamente qual COV é presente em cada uma das amostras; ele tem 32 sensores eletroquímicos que detectam padrões de ar exalado (cada sensor vai reagir com os COVs presentes no ar, gerando um sinal elétrico). Os dados desses 32 sensores são, então, reduzidos por análise do componente principal (PCA - *principal component analysis*) para os principais componentes de cada uma das coletas pelo próprio *software* do aparelho. Esses componentes do PCA foram utilizados para determinar padrões de ar exalado. Para este estudo, os perfis do ar exalado foram denominados padrão 1, 2 e 3.

### Considerações estatísticas

As considerações estatísticas foram feitas com base em estudos utilizando o Cyranose^®^ *320* em outras doenças pulmonares, estimando incidência de PAV de 35% no padrão 1 de ar e de 15% no padrão 2, para um poder de 80% e significância de 0,05, calculando-se amostra de 70 pacientes.

A análise primária foi realizada comparando o perfil do ar exalado entre os pacientes com e sem PAV. Para análise primária, foi considerado o primeiro ar coletado após a inclusão no estudo, o ar coletado nas 24 horas que antecederam o diagnóstico clínico de PAV e a mudança no perfil do ar entre esses dois tempos. Esse período foi selecionado tendo em vista a hipótese de que a mudança precoce no perfil do ar exalado pode prever o subsequente desenvolvimento de PAV. Para o Grupo Não PAV, foi considerado o ar coletado 96 horas após o início da ventilação mecânica para fins de comparação com o ar coletado nas 24 horas que antecederam o diagnóstico de PAV. Esse período foi selecionado para permitir comparação entre os perfis em tempo semelhante de ventilação mecânica no Grupo Controle (Não PAV).

Uma análise secundária foi realizada somente nos pacientes com PAV para determinar a capacidade do perfil do ar exalado prever desfechos clinicamente relevantes (tempo de ventilação mecânica e tempo de internação em UTI). Para tanto, além das coletas citadas anteriormente, foram utilizados dados do ar coletado no dia após esse diagnóstico. Assim, foi possível determinar não apenas o valor preditivo de coletas transversais do ar exalado, mas também o impacto da mudança temporal no perfil do ar exalado e sua relação com o desfecho.

As variáveis contínuas foram expressas como média e desvio-padrão (DP). Os dados categóricos foram expressos em frequências e porcentagens. Os grupos foram comparados por meio do teste de Kruskal-Wallis e do teste de Wilcoxon-Mann-Whitney, para desfechos contínuos, e do qui-quadrado, para desfechos categóricos, e regressão logística binária multivariada foi utilizada para analisar as diferenças ajustadas entre os grupos. Foram incorporadas no modelo todas as variáveis com p < 0,25 na análise univariada, além de idade, gênero, SAPS 3 e SOFA das primeiras 24 horas. Os resultados das regressões foram expressos como risco relativo (RR) e intervalo interquartil (IIQ); em todas as análises, foi adotado como nível para significância estatística p valor < 0,05 bicaudado.

## RESULTADOS

No período do estudo, foram avaliados 88 pacientes e, respeitando os critérios de exclusão predefinidos, foram inclusos 83 pacientes na análise final (Figura 1); entre eles, 38 (46%) apresentaram confirmação microbiológica. A mediana para o desenvolvimento de PAV foi de 4 dias, com intervalo 25 - 75 entre 4 e 6.

**Figura 1:** Participantes no estudo.

PAV - pneumonia associada à ventilação mecânica.

Na comparação entre o Grupo PAV e o Grupo Não PAV não houve diferença estatística entre as principais características dos pacientes no momento da inclusão: idade média de 60 anos (p = 0,29), predomínio do sexo masculino em ambos os grupos (p = 0,66), pacientes internados principalmente por motivos clínicos (p = 0,21), escore de gravidade SAPS 3 com pontuação média de 52,5 (p = 0,38) e escore de disfunção orgânica SOFA com pontuação média de 8,4 (p = 16) (Tabela 1). Na regressão binária, nenhuma dessas variáveis foi independentemente associada à ocorrência de PAV. Dentre as características sociodemográficas e clínicas dos pacientes que apresentaram os padrões 1, 2 ou 3 de ar exalado também não houve diferença quanto as variáveis analisadas (Tabela 2).

**Tabela 1:** Características dos pacientes no momento da inclusão no estudo

Variáveis	Grupo PAV (n = 23)	Grupo Não PAV (n = 60)	Valor de p	RR (IIQ)
Idade (anos)	58 ± 16	62 ± 15	0,29	
Sexo masculino	15 (65,2)	36 (60)	0,66	
Motivo da internação				
Clínico	14 (60)	30 (50)		REF
Cirurgia urgência	8 (34,7)	18 (30)	0,21	0,52 (0,15 - 1,7)
Cirurgia eletiva	1 (4,3)	12 (20)		1,43 (0,15 - 1,3)
Comorbidades				
Nenhuma	8 (34,7)	19 (31,6)		
Pulmonar	2 (8,6)	6 (10)	0,95	
Outras*	13 (56,5)	35 (58,3)		
SAPS	50 ± 18	55 ± 20	0,38	
SOFA D1	7,9 ± 2,6	9 ± 3,5	0,16	0,85 (0,71 - 1,01)

PAV - pneumonia associada à ventilação; RR - risco relativo; IIQ - intervalo interquartil; SAPS - *Simplified Acute Physiology Score*; SOFA - *Sequencial Organ Failure Assessment Score*; D1 = Dia 1. * Doenças cardiovasculares e diabetes. Resultados expressos como média *±* desvio-padrão ou n (%).

**Tabela 2:** Características dos pacientes em relação a cada padrão de ar encontrado nas primeiras 12 horas de admissão entre os Grupos PAV e Não PAV

Variáveis	Padrão 1	Padrão 2	Padrão 3	Valor de p
Idade (anos)	65 ± 15	57 ± 17	61 ± 12	0,89
Sexo masculino	18 (58)	21 (63)	12 (63)	0,88
Motivo clínico para internação	19 (61)	15 (45)	10 (52)	0,30
Comorbidades pulmonares	3 (9,6)	3 (9)	2 (10)	0,84
SAPS	53 ± 22	57 ± 18	48 ± 19	0,29
SOFA D1	8.5 ± 3,4	8.8 ± 3,1	8.8 ± 3,6	0,19

SAPS - *Simplified Acute Physiology Score*; SOFA - *Sequencial Organ Failure Assessment Score*; D1 - Dia 1. Resultados expressos como média *±* desvio-padrão ou n (%).

Os padrões de ar analisados nas primeiras 12 horas após a instalação da ventilação mecânica (ar admissão) foram preditores quanto ao desenvolvimento de PAV durante a internação (p = 0,018), mesmo quando submetidos à regressão binária (Tabela 3). Entretanto, a análise dos padrões de gases exalados 24 horas antes do diagnóstico clínico de PAV (ar pré-PAV) não foi preditor do desenvolvimento de PAV (p = 0,35). Em relação à mudança dos padrões de ar analisados ao longo da internação dos participantes, também não houve alteração significativa (p = 0,3) (Tabela 3). Em cada um dos dois tempos estudados (ar admissão e ar pré-PAV), assim como a mudança do padrão do ar encontrado em alguns pacientes, sensibilidade, especificidade e valor preditivo positivo para desenvolvimento de PAV apresentaram resultados pouco expressivos (sensibilidade e especificidade, respectivamente, de 30 e 60% no ar admissão para os padrões 2 e 3; sensibilidade e especificidade ambas de 60% no ar pré-PAV; e sensibilidade de 30%, especificidade de 75% com valor preditivo positivo de 39% para a mudança do padrão do ar).

**Tabela 3:** Relação entre os padrões de ar encontrados e o desenvolvimento de pneumonia associada à ventilação

	Grupo PAV (n = 23)	Grupo Não PAV (n = 60)	Valor de p	RR (IIQ)	RR (IIQ)*
Ar na admissão†					
Padrão 1	3 (13)	28 (46)		REF	REF
Padrão 2	13 (56)	20 (33)	0,018	8,05 (1,8 - 35)	6,8 (1,6 - 29)
Padrão 3	7 (30)	12 (20)		5,9 (1,2 - 29)	5,8 (1,2 - 27)
Ar pré-PAV‡					
Padrão 1	6 (26)	15 (25)			
Padrão 2	11 (47)	11 (18)	0,35		
Padrão 3	6 (26)	8 (13)			
Mudança§, sim	9 (39)	18 (53)	0,3		

PAV - pneumonia associada à ventilação; RR - risco relativo; IIQ - intervalo interquartil. * Ajustado na regressão binária; † amostra de ar analisada até 12 horas após inclusão; ‡ amostra de ar analisada 24 horas antes do diagnóstico clínico de PAV; § mudança do padrão de ar durante a internação dos participantes. Resultados expressos como n (%).

O tempo médio de ventilação mecânica e o número de dias de internação em UTI foi maior no Grupo PAV, conforme esperado. O Grupo PAV apresentou, em média, 13 dias de ventilação mecânica comparado a 5 dias do Grupo Não PAV (p = 0,0001), o mesmo ocorrendo com os dias de internação na UTI, sendo 17 dias em média no Grupo PAV e 9 dias no Grupo Não PAV (p = 0,0001). Apesar disso, o padrão do ar exalado coletado na admissão não teve relação com tempo de VM (p = 0,28) e nem com os dias de internação na UTI (p = 0,40). Isso também se repetiu para o ar pré-PAV (p = 0,40 e p = 0,41) e para o ar após o diagnóstico de PAV (p = 0,58 e p = 0,23). Também não houve correlação estatística entre um determinado patógeno e um padrão específico de ar exalado.

## DISCUSSÃO

No presente estudo, determinamos que o padrão de ar exalado quando coletado nas primeiras horas após a instalação da ventilação mecânica é capaz de predizer a ocorrência de PAV.

O ar pré-PAV (ar analisado durante as 24 horas que antecederam o diagnóstico de PAV) não foi capaz de predizer PAV conforme esperado pelos autores do estudo. Entretanto, a análise das amostras de ar das 12 horas da admissão (ar admissão) foi capaz de predizer o diagnóstico de PAV. Isso pode ser explicado pelo fato de o desenvolvimento da PAV ter relação com fatores intrínsecos do próprio indivíduo, ou seja, algumas características próprias da população estudada, não identificadas neste estudo, foram capazes, por si só, de predizer PAV. Dentre essas características, deve-se destacar o grau de inflamação, a qualidade e a intensidade do estresse oxidativo presente na mucosa respiratória no momento da inclusão desses participantes. É possível que essa alteração precoce do ar admissão seja também justificada pelas condições ou pelos motivos pelos quais o paciente foi submetido à intubação. Uma intubação realizada de maneira não adequada, tardia ou pós-broncoaspiração por exemplo, em teoria, pode alterar, de forma precoce, a mucosa das vias aéreas. Em trabalhos futuros, seria importante essa correlação entre o motivo imediato da intubação orotraqueal, se eletiva ou de urgência, e as análises temporais do ar exalado. Este resultado também não foi, de alguma maneira, influenciado pelas características próprias dos indivíduos (idade, sexo, comorbidades, motivo de internação, SOFA, SAPS 3 e valor de proteína C-reativa [PCR]) quando são distribuídos em relação aos padrões 1, 2 ou 3 de ar estudados, pois não houve diferença entre essas variáveis entre os três grupos. Os COVs são frutos do estresse oxidativo presente no epitélio da arvore respiratória, e essa inflamação pode ser facilitadora para o desenvolvimento de PAV nesse grupo de pacientes.^15^

Outro resultado encontrado corroborado por esta discussão foi a não mudança do padrão do ar ao longo do acompanhamento dos participantes. Observou-se que, nos pacientes que desenvolveram PAV, o padrão do ar permaneceu semelhante desde o início da inclusão. Algo adquirido durante a internação (exemplos: colonização de determinados microrganismos já presentes no sistema respiratório, episódios de microaspirações e outros fatores de risco já conhecidos na literatura), que até pode ter relação no desenvolvimento da PAV, não parece influenciar, pelo menos em nossos pacientes, a qualidade do ar analisado. As características inflamatórias próprias do epitélio respiratório dos participantes, já no momento da inclusão, podem ter sido responsáveis pela predição do ar admissão no diagnóstico de PAV.^(16)^

Durante todas as análises do ar realizadas ao longo do trabalho, o aparelho foi calibrado e treinado a partir do ar ambiente; essa maneira de avaliação pode ter influenciado nos resultados. O Cyranose^®^ 320 é uma ferramenta que possui a capacidade de aprender a detectar odores a partir de uma memória armazenada em seu *software*. Essa funcionalidade permite o uso do aparelho na detecção de odores específicos ou semelhantes em várias áreas da saúde, como na detecção precoce do câncer colorretal e no auxílio diagnóstico de algumas patologias do trato respiratório, como na asma e na doença pulmonar obstrutiva crônica.^(4,17)^ Há possibilidade, caso o aparelho seja treinado a partir de amostras de ar de indivíduos já com o diagnóstico firmado de PAV e com ar de cepas de micro-organismos mais comuns em unidades de terapia intensiva, de que os resultados sejam mais preditores e que se identifique de uma maneira mais refinada uma mudança do padrão de ar, a ponto de caracterizar um diagnóstico precoce de PAV. Essa hipótese pode justificar o motivo de não ter ocorrido mudança significativa do padrão de ar durante a evolução dos casos que desenvolveram PAV, pois o aparelho não estava treinado (calibrado) para detecção específica de odores semelhantes de pacientes com diagnóstico de PAV.

O ar da admissão foi capaz de predizer PAV, entretanto não foi eficaz para predição de gravidade no Grupo PAV. Esperava-se que, no Grupo PAV, a mortalidade também fosse maior que no Grupo Controle, e isso não aconteceu, pois a taxa de mortalidade foi semelhante entre os grupos.^(18,19)^Consequentemente, nessa população estudada, não se mostrou relação direta entre PAV e gravidade. Isso se deveu, provavelmente, pelo pequeno tamanho da amostra alcançada no Grupo PAV. É possível haver uma relação direta entre o ar analisado como preditor de PAV e esse mesmo ar predizer gravidade em uma amostra populacional maior. Essa mesma justificativa, de um tamanho amostral reduzido presente na pesquisa, pode ser usada para explicar as baixas sensibilidade e especificidade e o valor preditivo positivo encontrados nos resultados.

De forma semelhante, tanto o ar admissão quanto o ar pré-PAV não foram capazes de diferenciarem PAV precoce e PAV tardia. Isso pode ser decorrente da pequena amostra obtida no Grupo PAV e, também, pela calibragem do aparelho ter sido feita em relação ao ar ambiente. É possível que, em aparelho treinado e calibrado em amostras de ar de indivíduos com PAV, haja algum resultado em predição quanto à classificação temporal da PAV (precoce ou tardia).

Os resultados encontrados neste estudo encorajam a produção de outros trabalhos científicos com o uso do nariz eletrônico para o diagnóstico de PAV e seus determinantes microbiológicos. Já existem estudos demonstrando a importância da análise do ar exalado em algumas infecções pulmonares, ou seja, na identificação do micro-organismo responsável pela infecção.^(20)^ A suspeição do germe causador da PAV à beira leito possibilita o tratamento antimicrobiano precoce e minimiza o uso indiscriminado de antibióticos durante a espera pelos resultados de culturas de secreções respiratórias.

Apesar disso, algumas limitações do estudo devem ser levadas em conta, ao se analisarem os resultados. Primeira, uma série de pacientes, com patologias comuns em terapia intensiva, foram excluídos, o que reduz a validade externa do estudo. Apesar de ser uma limitação, com isso temos uma amostra de pacientes mais homogênea, que talvez possa refletir de maneira melhor somente a influência da ventilação mecânica no desenvolvimento de PAV e minimizar o efeito de outras doenças sobre o perfil do ar exalado. Segundo, por limitações de tamanho de amostra e característica da variável preditora (categórica), a construção de uma curva *Receiver Operating Characteristic* (ROC) e sua interpretação são limitadas, dificultando uma interpretação mais robusta do real valor preditivo da análise do ar exalado no diagnóstico de PAV. Terceiro, existe uma diferença importante na definição epidemiológica utilizada aqui para o diagnóstico de PAV e o critério clínico para o início de antibiótico. Assim, alguns pacientes que, na prática, seriam incluídos como portadores de PAV, não o foram no presente estudo. O uso da definição epidemiológica, por outro lado, deixa o Grupo PAV mais homogêneo, o que pode ser importante para o objetivo primário do estudo e permitir comparações e análises de resultados futuros com outras pesquisas que utilizaram esse mesmo critério definidor.

## CONCLUSÃO

A utilização do aparelho nariz eletrônico Cyranose^®^ *320*, por meio da análise do ar exalado durante as primeiras 12 horas do uso da ventilação mecânica invasiva, foi capaz de identificar uma relação com o desenvolvimento evolutivo de pneumonia associada a ventilação mecânica na população estudada. Entretanto, as diversas amostras de ar captadas pelo nariz eletrônico não foram capazes de predizer gravidade e nem identificar o desenvolvimento de pneumonia associada *à* ventilação mecânica precoce ou tardia entre os participantes.
